# Patient Safety during ECMO Transportation: Single Center Experience and Literature Review

**DOI:** 10.1155/2021/6633208

**Published:** 2021-02-22

**Authors:** Mateusz Puslecki, Konrad Baumgart, Marcin Ligowski, Marek Dabrowski, Sebastian Stefaniak, Malgorzata Ladzinska, Ewa Goszczynska, Pawel Marcinkowski, Anna Olasinska-Wisniewska, Tomasz Klosiewicz, Aleksander Pawlak, Marcin Zielinski, Lukasz Puslecki, Roland Podlewski, Lukasz Szarpak, Marek Jemielity, Bartlomiej Perek

**Affiliations:** ^1^Department of Medical Rescue, Poznan University of Medical Sciences, Collegium Adama Wrzoska Rokietnicka Street 7, Poznan 60-806, Poland; ^2^Department of Cardiac Surgery and Transplantology, Poznan University of Medical Sciences, Dluga Street 1/2, Poznan 61–848, Poland; ^3^Polish Society of Medical Simulation, Slupca, Poland; ^4^Department of Medical Education, Poznan University of Medical Sciences, Collegium Adama Wrzoska Rokietnicka Street 7, Poznan 60-806, Poland; ^5^Voivodeship Emergency Station, Poznan, Poland; ^6^Department of International Management, Poznan University of Economics and Business, 10 Niepodleglosci Av., Poznan 61-875, Poland; ^**7**^ Sklodowska-Curie Medical Academy, 12 Solidarnosci Av., Warsaw 03-411, Poland

## Abstract

**Background:**

Extracorporeal membrane oxygenation (ECMO) has been proven to support in lifesaving rescue therapy. The best outcomes can be achieved in high-volume ECMO centers with dedicated emergency transport teams.

**Aim:**

The aim of this study was to analyze the safety of ECMO support during medical transfer on the basis of our experience developed on innovation cooperation and review of literature.

**Methods:**

A retrospective analysis of our experience of all ECMO-supported patients transferred from regional hospital of the referential ECMO center between 2015 and 2020 was carried out. Special attention was paid to transportation-related mortality and morbidity. Moreover, a systematic review of the Medline, Embase, Cochrane, and Google Scholar databases was performed. It included the original papers published before the end of 2019.

**Results:**

Twelve (5 women and 7 men) critically ill ECMO-supported patients with the median age of 33 years (2–63 years) were transferred to our ECMO center. In 92% (*n* = 11) of the cases venovenous and in 1 case, venoarterial supports were applied. The median transfer length was 45 km (5–200). There was no mortality during transfer and no serious adverse events occurred. Of note, the first ECMO-supported transfer had been proceeded by high-fidelity simulations. For our systematic review, 68 articles were found and 22 of them satisfied the search criteria. A total number of 2647 transfers were reported, mainly primary (90%) and as ground transportations (91.6%). A rate of adverse events ranged from 1% through 20% but notably only major complications were mentioned. The 4 deaths occurred during transport (mortality 0.15%).

**Conclusions:**

Our experiences and literature review showed that transportation for ECMO patients done by experienced staff was associated with low mortality rate but life-threatening adverse events might occur. Translational simulation is an excellent probing technique to improve transportation safety.

## 1. Introduction

The Extracorporeal Life Support Organization (ELSO, Ann Arbor, MI, USA, http://www.elso.org) is a worldwide nonprofit patronage organization of more than 900 centers units providing almost 200,000 ECMO per year [1]. The main mission of ELSO is to collect data on ECMO applications and develop guidelines regarding all aspects of therapy, including transport of EMCO-supported individuals [2].

ELSO recommends creation of centers dedicated to treat patients by means of sophisticated extracorporeal techniques, including ECMO. Due to its complexity, outcomes were shown to correlate with center volume. Therefore, the minimum number was set at the level of 6 applications per year [3–8]. The optimal number that has been linked to significant reduction in mortality is at least 30 adult supported individuals per year. As a consequence, consolidating of ECMO therapy in the specialized large-volume centers has been strongly recommended. Such centralization has introduced a highly specialized patient transport between referring hospitals and reference centers. This model has been adopted around the world and has been confirmed as save and associated with the optimal outcomes [3, 4, 9, 10].

The main aim of this study was to assess the safety of patients transported during the use of extracorporeal techniques on the basis of our experience as a referral hospital for ECMO-treated patients, a coordinating body in innovation and cooperation, as well as a systematic review of previously published clinical reports.

## 2. Methods

### 2.1. Analysis of Our Experience

We analyze the safety of ECMO patients transferred from the regional hospitals to our ECMO center in the last five years. To prepare for the first real life transport by conventional emergency ambulance, we performed simulation scenario that involved a case of refractory respiratory failure in one city approximately 80 km from referential ECMO center. It was blinded for physicians and paramedics engaged in on-site intervention, transportation, and admission to destination hospital. All weak points were found, analyzed, and discussed in detail and actions to improve safety have been developed.

### 2.2. Transfer of Patients

Analysis of transfer of our ECMO-supported patients, including patients' basic demographic variables, indications and modes of support (venovenous or venoarterial), and transportation characteristics, was performed. Special attention was paid to “in transportation” safety, including mortality and morbidity.

### 2.3. Data Management

As continuous variables such as age of studied patients and length of transfer have not satisfied criteria of normality (estimated by means of the Shapiro-Wilk test), they were presented as the medians with ranges (minimal through maximal values).

### 2.4. Systematic Review

A systematic review of the Medline, Embase, Cochrane, and Google Scholar databases was performed to find articles reporting primary or secondary ECMO transportation. Studies were included if they described medical or technical complications during interhospital transfers of patients with extracorporeal support. It included the original papers published before the end of 2019 and the search string was comprised of the following MeSH (Medical Subject Headings) and Booleans: ((“Extracorporeal Membrane Oxygenation”[MeSH] OR “ECMO”[MeSH]) AND (“Transportation”[MeSH] OR “Patient Transfer”[MeSH])) AND “critical”[MeSH Terms] AND “adult”[MeSH Terms] and “safety”[MeSH Terms].

In addition, the following inclusion criteria including papers in English, published after 2009 in the form of randomized controlled trials (RCTs), meta-analyses, practice guidelines, and reviews were used. Articles dealing with the outcomes of exclusively pediatric and neonatological transfers, conventional transports without ECMO support, and the case studies were excluded. Three investigators reviewed all articles, performed data extraction, and compiled the database using equal digital templates (MP, KB, and MD). The search results were checked for the title and content of the abstract; then, the full text for review was obtained. Only the latest publications from a reporting center were included in the study. Results were compared, and discrepancies were solved by agreement. Duplicated results were obviously excluded. A fourth investigator approved the final database and decided upon remaining conflictive data (BP). In case of suspected unreliable information due to insufficient clarity in data presentation, a given article was excluded from the analysis or was solved by consensus of the research team. The process followed the PRISMA guidelines for systematic reviews and meta-analyses and the Cochrane guidelines for systematic review of interventions.

Once appropriate articles were allocated, the following general study and technical information were collected: center and country of realization, year of study start and year of its completion, reported period, number of transportation, primary/secondary transfer, vehicle type, deaths or adverse events, minimal and maximal transfer length, type of ECMO support (venovenous or venoarterial), and number of ECMO team persons. Patient deceases were considered to be attributable to complications during ECMO transportation whenever authors of the original article clearly identified a direct relation between the occurrence of the complication and the fatal outcome. Such complication must have occurred during the transfer and not related to cannulation.

A critical analysis of all aspects of transportation with ECMO support was made. In addition, attempts were made to identify the key components of this process that affects the patient's safety [11–32].

## 3. Results

### 3.1. Primary Simulation Scenario

This has previously been described in much detail in earlier authors' publication [32]. A video-film was recorded and carefully analyzed by all members involved in cooperation of mobile ECMO team. The purpose of the created high-fidelity scenario was to verify the ECMO transportation procedure created for the “ECMO for Greater Poland” program. The simulation tested the communication and collaboration of several medical teams in prehospital and hospital settings. The specific objectives of the simulation were conducted to assess critical points and compiled into the scenario checklist for the ECMO transportation algorithm. That “probing simulation” creates transportation procedure for our dedicated mobile ECMO team to set standards for the future real-time/real-life ECMO transportation.

### 3.2. Our Experience

Twelve critically ill ECMO-supported patients (5 women and 7 men) with the median age of 33 (2 to 63) years were transferred to our ECMO center. 91% (*n* = 11) of our patients were in the venovenous (VV) mode with the remainder 8% being venoarterial (VA). The median transfer length was 45 km and ranged from 5 to nearly 200 km. In all cases, mobile ECMO team consisted of 4 of 5 members representing critical paramedics (1 or 2), perfusionist, cardiac surgeon, and specialist in intensive care. A number of professionals involved in transportation depended on ambulance type and in ECMO-dedicated vehicles there were was 5 persons whereas there were 4 in conventional ambulances.

The predominant indication for mobile ECMO team activation was deteriorating respiratory failure, refractory to conventional therapy with mechanical ventilation (11 cases). Preferable technique for cannulation was the percutaneous approach and only in one case surgical exposure of the peripheral vessels was carried out. One VA support and subsequent transportation were done when the patient was connected to intra-aortic balloon pump (IABP) that had been inserted a few hours earlier. In our group, nobody died and no adverse events were observed. The details are summarized in Table 1.

### 3.3. Literature Review

**Figure 1:**
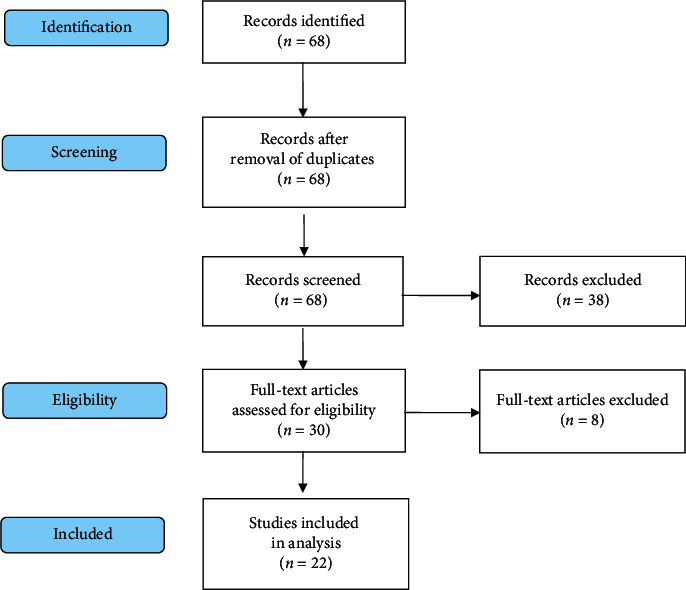
Research flowchart according to PRISMA statement.

In total, 68 publications were found and at the very beginning of study selection 38 of them was excluded because they did not meet the established criteria (they included only pediatric or neonatological transports; their full text was not published in English or accepted as Letters to Editor). Thirty full text articles satisfied the search criteria; thus, they have been reviewed. Another 8 papers were discarded after detailed analysis due to type of reports (case studies) or because they did not include any ECMO transfer data (transfer type, transfer vehicles, distances, and adverse events). Eventually, 22 articles were analyzed, as shown in Figure 1.

A total number of 2647 transfers were reported. Among them, in 8 publications, more than 100 patient transportations were described. They were predominantly primary transfers (90%) and ground transportations (approximately 91.6%). Of note, various means of transport such as ambulance, helicopter, plane, or hybrid transfer (ambulance + helicopter, ambulance + plane, ambulance on the plane) were employed. The median number of reported transfers per month was 1.2. The median of minimal distance was 5 km (0.5–126) and the median of maximal one was 225 km (25–11398.2). Although all teams had been prepared to apply either VV or VA support, the vast majority of critically ill patients required VV support. The number of team members ranged from 2 to 6 people (median 3) (Table 2).

In the authors' analysis, only 15 deaths out of 2647 adult patient transfers were reported in the years 2013 to 2019 [12, 13, 23, 25, 28, 31]. In that group, there were 11 deaths associated with cannulation but not directly to transportation and only 4 (0.15%) were directly related to medical transfers to referential centers; see [12, 25, 31]. In the Bryner series published in 2014, 5 patients died (mortality rate 2.3%) before transport and one (0.5% mortality) died during preparations for takeoff of the fixed-wing aircraft [12]. Another death reported by Brechot et al. [25] was caused by significant hemodynamic deterioration during transport. Two last deaths were reported in Bromar et al. publication and in next report from Karolinska center by Fletcher-Sandersjoo et al. The mortality rate was 0.15% of transports of ECMO-supported patients.

Other described procedural complications did not affect survival. The adverse events rates have been found in the wide range from 1% to even up of 20% according to Fletcher-Sandersjoo et al.

Austin et al. reported technical problems in 7.3% transfers that included ECMO pump power failure (*n* = 3), ventilator (*n* = 1) or infusion pump failure (*n* = 1), and oxygen depletion (*n* = 1). Four patients required electrotherapy at the time of transfer (pacing = 3, cardioversion = 1). Tamponade of the pericardium and aeration of the system were noted twice. The original cannulation strategy was changed twice. The most common problems were hypothermia (*n* = 8), hypotension (*n* = 11), and hypoxia (*n* = 17) [26].

Sherren et al. described one case of recirculation (2.0%) and one case of death at the initiation of therapy in the reporting center (patient with multiple organ failure and history of multiple cardiac arrests). Additionally, hypoxia, hypotension, and arrhythmias classified as low-order adverse events were sporadically observed (only in 31% of transfers) [13].

Ehrenhaut et al. recorded death before departure (*n* = 4) and cardiac arrest before departure followed by return of spontaneous circulation (*n* = 4). Additionally, they reported 15.6% complications including incorrect position of the cannula (*n* = 7) and problems with identification of the peripheral vessel (*n* = 7). They also reported 6.3% high-risk complications such as hemodynamic instability (*n* = 1), bilateral tension pneumothorax (*n* = 1), and another problem with ventilation (*n* = 1). Two incidents occurred while driving [28].

Vaja et al. reported (1.0% complications) severe arrhythmias (ventricular tachycardia) during cannulation followed by return to spontaneous circulation [14].

Blecha et al. distinguished three sources of events related to total 431 transfers (52 on ECMO, 379 conventional without extracorporeal support):

  Patient: this included cardiac arrest (*n* = 3), accidental extubation (*n* = 2), hypoxia (*n* = 30), and hypotension (*n* = 7)
   Technical items: these included mainly problems with the respirator (*n* = 3)
   Means of transport: this included a need to change the ambulance (*n* = 3) and unplanned delays (due to staff, weather, and others). No high-risk complication in ECMO support transfers was reported [22]

Guenther et al. listed the wrong position of the cannula (*n* = 1) and the failure of invasive blood pressure monitoring (*n* = 1) [19].

Heuer et al. noticed sudden reduction in the ECMO flow due to hypovolemia [24]; Salna et al. marked one incident of partial pressure changes associated with high attitudes [16] and 7.6% of patients in Brechot group manifested significant hemodynamic deterioration during transport [25]. The other authors did not report high-risk and life-threatening complications [11, 15, 17, 18, 20, 21, 23, 27, 29, 30, 32].

## 4. Discussion

### 4.1. Patients' Volume

In the ELSO guidelines recommending the development of ECMO centers, the rationale for strategy was reports showing the centers performing over 20–30 therapies per year that had clearly better outcomes. By creating ECMO centers according to the health needs of the population and accumulating both sufficient patients and experienced staff in these centers, the effectiveness of this challenging therapy is expected to be better [33–37]. However, there are no available data regarding minimal number of ECMO transports to be performed by the mobile teams to gain mandatory experience. Most publications included small cohorts of transported patients. Only in sixteen of them, a number of transports were exceeding 50, with 11 above 100. Of note, in one Swedish center, their number exceeded 900 [25]. In our study, only 12 patients have been transported between hospitals in the few last years. However, our experience gained in the cardiac surgery department regarding cannulation of the peripheral veins and arteries for extracorporeal circulation and security measures developed for in-hospital transport has resulted in the favorable results of ECMO-supported patients transfer, in spite of relatively low number of transportations.

### 4.2. Hub and Spoke

In order to achieve early and late optimal outcomes, it is necessary to bring the patients to centers with comprehensive and advanced treatment. The concept of “HandS-Hub and Spoke” was introduced by Combes et al. [33]. Safe patient transfer is a necessary bridge between ECMO centers and reference hospitals specialized in the conventional therapies, but also those that can initiate ECMO support [2]. The availability of beds will depend on capacity, mode of transport supported (ground and air), physical and geographical location, and the number of inhabitants of a given area. Patients who require ECMO support can be transferred to a large-volume center by a mobile ECMO team. Bearing in mind the risk of seasonal viral infections such as AH1N1 influenza and SARS-Cov-2, particularly in the aging societies with comorbidities, a temporary need for the use of this aforementioned support is important. Broman et al. suggested that, for the treatment of respiratory failure among the three age groups (neonatology, children, and adults), one ECMO center per 5–10 million population should be prepared whereas for the adults only one should be prepared per 8–15 million, respectively [34].

The international experts have suggested that transport of unstable refractory respiratory patient is safer when being on ECMO than when being conventionally ventilated [35, 36]. Brechot et al. in a retrospective single center study that involves 118 transfers confirmed the safety of patient ECMO transfer. Moreover, the results of these patients were comparable with the outcomes of patients in whom ECMO support was carried out in the ECMO center, without any transfer [25].

The authors of this article have developed a pioneer regional ECMO program in Poland: “ECMO for Greater Poland” in 2016. The intention was to create algorithms and improve the coordination of Medical Rescue Teams in the “ECMO rescue chain.” Previous simulation-based training allowed building a successful procedural chain and then eliminating errors at various stages in identification, notification, transportation, and ECMO support [32].

### 4.3. Transport Definition

It is a combination of classical transfer of critically ill persons (suffering from failure of at least one organ or system), often mechanically ventilated, receiving continuous infusion of drugs and/or treated by means of extracorporeal circulation (ECC). Transporting them during ECMO support is very demanding due to the high complexity, chronic shortage of time, and environmental pressures. Expert consensus indicates that each ECMO network should have an appropriate ECMO Mobile Team [33, 38].

The “ground time,” the period of necessary resuscitation/stabilization and careful observation after ECMO support initiation, switching over to the transport unit's equipment, and before leaving the reporting unit, may last up to 5 hours [39]. A special attention must be paid to identify any potential problems that may appear during transportation. ELSO has already issued guidelines for transportation to assist in the most appropriate vehicle selection [2]. The time from the initial qualification of any patient by ECMO physician to the bedside assessment at the call point should be as short as possible [18].

A primary transport is defined if the mobile ECMO team performs the cannulation of the patient and initiation of therapy at the referring hospital and then supervises transfer to an ECMO center [2,37]. A subtype of primary transport is when the mobile team initiates ECMO support at the regional hospital, but placement of the cannulae is performed by the referring to hospital's physicians. A transport is defined as secondary if the patient had already been on extracorporeal support but the mobile ECMO team supervised only transfer to the Center of Excellence [39].

### 4.4. Transportation Vehicle

#### 4.4.1. Ground Transport

It is a complex logistics task to plan, control, and implement optimal transport strategy (in ELSO recommendation, up to 400 km (250–300 miles)) [2]. When properly prepared, it should be repeatable and simple to carry out. All centers included in the study used the land route. For the purpose of ECMO transport, it is necessary to equip the vehicle with an efficient 230V installation, capable of securing the power supply of electrical devices with a safe power reserve. Above-standard and energy-consuming devices such as the ECMO pump and heat exchanger should be taken into account. It is also necessary to provide a supply of medical gases capable of covering the needs of mechanical ventilation devices and an oxygenator. This requires prior planning of the length and time of the journey, assuming that the gas reserves should cover twice the time estimated for the transport. The patient can usually be accessed from at least three sides. Equipment can be attached to stretchers, handles, or mounting rails installed in the medical compartment. Having in mind all aforementioned requirements of optimal ECMO ground transport, members of our team actively participated in equipping of the special ambulance dedicated for critically ill patients. Possessing such ambulance, our team felt comfortable; thus, all our transfers were done by ground transport.

**Figure 2:**
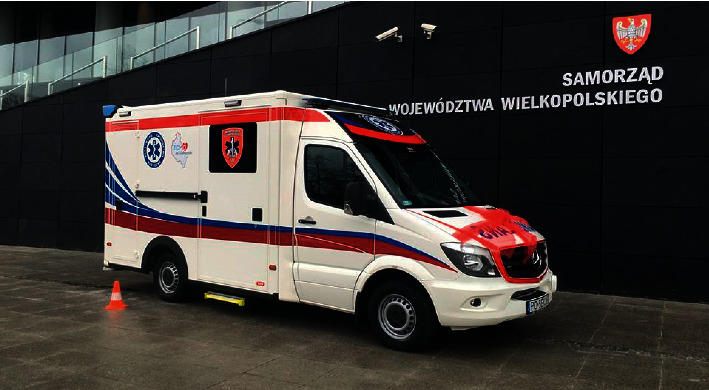
Ambulance used to transport critically ill patients in “ECMO for Greater Poland” program.

The key requirements of mobile intensive care units (MICU) equipment are difficult to standardize and they differ from country to country. Typically, it is a C-type ambulance adapted to carry more medical devices, equipped with additional power supply, ventilation, suspension, and a patient transport system, as shown in Figure 2. With regard to the current conditions in the Republic of Poland, there is no systematic mobile intensive care program, and the availability of this type of transport with a qualified team is negligible and depends on the internal arrangements of ECMO centers [32]. Yeo et al. reported the possibility of transport without the use of an advanced respirator, replacing it with a self-inflating bag. Ventilation was monitored with a portable device for analyzing critical parameters [18].

#### 4.4.2. Air Transport

The use of air transport depends on its availability (the aviation team is in the hospital structure, having a hospital landing pad, local medical air transport policy, etc.) and terrain obstacles (ELSO recommendation for distances up to 650 km or 300–400 miles). Some centers have developed fixed-wing aircrafts transfers, mostly for international transportation [2]. Thirteen studies reported flights by airplanes, especially those used for long-distance transports [12–16, 19, 22–24, 26, 28, 30, 31]. Other centers have employed a “hybrid” transfer; the ambulance is placed inside a military plane, which allows it to avoid potentially risky displacement of the patient [30].

Air transportation may have the significant and unfavorable effect on the vital parameters of the patient, such as heart rate, systemic arterial pressure, and intracranial and stomach pressure [31]. Additionally, air missions are associated with exposure of both patient and medical staff to the 8 classic stressors such as hypoxia, changes in barometric pressure, temperature variations, reduced air humidity, noise, vibrations, and easier to tire and gravitational forces (overloads). The risk is associated with the occurrence of hypoxia and hypothermia resulting from the drop in atmospheric pressure and an increase in the volume of gas in physiologically aerated (e.g., sinuses) or not (e.g., pneumothorax, free air behind the eyeball) body spaces or equipment (intubation/tracheostomy tube cuff). However, taking into account the cruising altitudes of helicopters, the aspects related to pressure changes are irrelevant, and the hermetic cabin of the medical plane makes the issues of pressure changes negligible. Additionally, the forces of inertia (acceleration/deceleration) and vibrations of varying degrees are active [13–18, 31].

Furthermore, this transport mode does not always guarantee time savings (time-consuming organization) and thus should be used only on long and very long routes [12]. It can be also indispensable when moving the patient over difficult terrain or from places surrounded by water. The helicopter must use dedicated hospital landing pads and airports. Moreover, there is limited space for equipment and personnel which makes in-flight interventions much more difficult, even impossible (e.g., endotracheal intubation and full inspection of the ECMO system). There are also needs for a landing pad and bringing the patient to the airport with additional transport by ambulance required. Repeatedly moving patients from one point to the next with proper transfer protocols also poses possible risks such as inadequate supervision monitoring and timely interventions, as well as reliance on limited battery power and cylinder gases. Moreover, the economic aspects of such missions should also be taken into account.

#### 4.4.3. ECMO Mobile Team

None of the guidelines have defined the minimum personnel composition of the mobile ECMO team and therefore each of the centers developed its optimized concept [2]. Depending on the legal and organizational conditions of a given country, different competences, duties, and local traditions, the number of its members can be different. Mobile team should be capable of professionally treating a patient with critical cardiopulmonary failure refractory to conventional therapy. Additionally, the availability of such services should be possible at all times. The relevant scope of competence should include experience in critically ill patient transportation, skills in cannulation, and ECMO system support as well as patient care.

There are general recommendations for qualified medical professionals including physicians, transport specialists, nurses, perfusionists, and other ECMO specialists [4]. When analyzing the selected scope of literature in terms of the personnel involved in transport, in Bonn model, the minimal number of staff is two and consists of anesthetic nurse and anaesthesiologist [28]. It should be noted that in this case the ambulance (driver-rescuer and lifeguard) or the aircraft team (commander, pilots, and others) tasked to handle transport and equipment does not constitute the ECMO team per se. The ECMO Karolinska Transport Service team usually includes 3 to 4 persons [33] including the ECMO physician, intensive care nurse with ECMO competences (called ECMO specialist), and surgeon with experience in adult and pediatric thoracic surgery. The operating nurse may join such team for international transports as decided by the surgeon. Ericsson et al. proposed abandoning the models with strictly defined roles so as to optimize effectiveness and safety. The ECMO specialist and physician should have full competence and authorization to operate the system and its components [39]. In most centers (including ours), the ECMO circuit is primed by a perfusionist but in the other centers this may be accomplished by physician or nurse after completion of education courses. Depending on center preferences, the cannulation is performed through surgical access or percutaneously [11–33].

Mobile ECMO team developed in “ECMO for Greater Poland” program consists of two critical paramedics, perfusionist, cardiac surgeon, and intensivist. That team is prepared to evaluate the patient and ECMO indication, introduce ECMO cannulas to the dedicated vessels, perform prime of the extracorporeal device, and initiate and manage of all aspects of critically ill patient treatment, including the ECMO circuit, ventilation, medications, and anticoagulation. Additionally, they are trained and prepared for urgent intervention to resolve possible and not uncommonly life-threatening complications [32].

#### 4.4.4. Equipment

Some of the devices including anticoagulation monitoring (i.e., ACT analyzer) and additional surgical equipment (sterile instruments, sutures, dressing, and head lamps) or transportable ultrasound should be secured by self-sufficient mobile ECMO team. Additionally, it is recommended to double the sterile equipment because they are usually not available at the referring hospital. Obviously, manual or backup power ECMO supply, spare emergency ECMO circuit, connectors, extra pump head and oxygenator, and spare cannulas should be provided.

**Figure 3:**
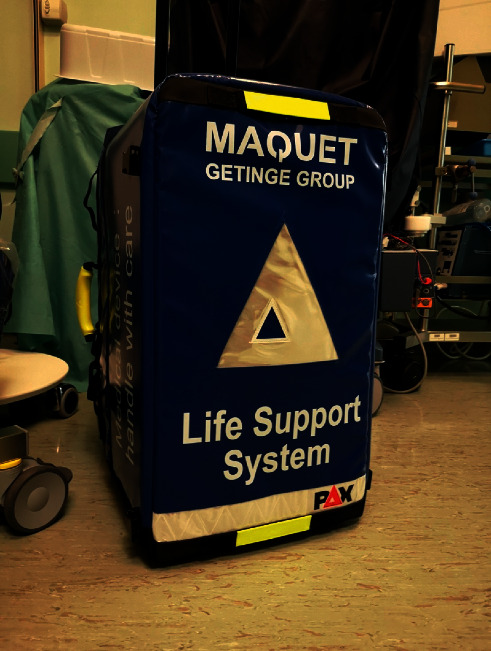
Transport bag for ECMO device.

To save time from activation to departure for the patient, the teams use preprepared kits signed bags that are regularly checked for completeness and expiry dates, as shown in Figure 3.

Equipment verification is done using a checklist to avoid missing essential items. The team's equipment proposal is included in Appendix 1 in Supplementary Materials. However, the autonomy of a given center in terms of equipment is recommended [2, 3, 39, 40].

Mobile ECMO teams maintain their readiness round the clock, often with varying lengths of response time during the day and during the night. Taking into account the key aspects of reaching the qualified bedside assessment as quickly as possible, transport teams regularly check the completeness and efficiency of the equipment elements.

#### 4.4.5. Checklists

A standard operating procedure (SOP) is a set of established steps and rules that must be followed until a desired state of affairs is achieved. It gives control over the course, supports situational awareness, and prevents overlooking important elements. SOPs have their source in the aviation community and prehospital medicine and battlefields are drawn from them. Standardized equipment preparation and sequence of operations are crucial in avoiding human error by improving intrateam communication and patient safety [41]. From the psychological perspective, demanding and stressful circumstances may significantly disturb situational awareness [42]; hence, there is a necessity to provide help in the forms of checklists. Emergency procedures without purpose and plan put unnecessary risk on both professionals and patients [43–45].

The procedures and checklists are divided into (a) clinical key actions taken before and during the transfer and (b) hardware/logistics such as equipment, notification algorithms, and organization. Checklists and standard procedures were adapted from aviation, where there was a panacea for increasing crew requirements and increasing complexity of aircraft. The case of Elaine Bromiley in 2005, who was severely mutilated during induction for routine surgery as a result of a series of crucial actions, is also often mentioned. Her husband (a professional pilot) decided to promote among the medical professions a procedural approach adopted from aviation [43, 44]. A breakthrough moment for modern medicine was the introduction by the International Health Organization, the perioperative control card, which significantly reduced the number of complications in the operational fields [45].

In the concept phase of the “ECMO for Grater Poland,” a review of the literature and available materials published by the other experienced ECMO centers were included. On their basis, in combination with high-fidelity simulations [32], an owned adaptation of checklists and hardware as the result of innovation cooperation of all parties involved, presented below, was developed. During the summary, SOPs were constantly evaluated and adapted to the changing needs, requirements, and possibilities. The following innovation procedures and checklists have been developed for the ECMO transport team:

1. “Prequalification card”: an idea of creating this card is to provide the ECMO coordinator for analysis with the necessary diagnostic data (e-mail and fax) in order to identify the need for extracorporeal therapy. Additionally, it contains the basic equipment and logistic requirements recommended in the reporting center (access to gas, electricity, etc.).
2. Procedure “arrival”: this is procedures for the ECMO and regional Emergency Medical Service (EMS) coordinators that support the decision-making and logistic process of transport organization.
3. Checklist “equipment”: this is the list of necessary equipment to be completed and reviewed prior to departure to the reporting center that is divided into “perfusion” and “intensive” equipment.
4. Procedure “with the patient”: it presents the steps that are taken by ECMO coordinator such as bedside assessment and final decision on possible ECMO therapy and its type (VV and VA), by nursing and ECMO team. The purpose of this document is to provide the appropriate forces and resources and to maintain the correct sequence of actions.
5. Procedure “departure with the patient”: this is the most demanding of the stages. A role of this document is to pay close attention to the division of roles of team members involved in cooperation to secure energy and gas reserves and to reach safely the means of transport.

#### 4.4.6. Outcomes

In our systematic review, only 15 deaths out of 2647 adult patient transfers were reported,11 deaths associated with cannulation but not directly to transportation and only 4 (0.15%) directly related to medical transfers. Well-prepared and experienced teams guarantee safe long- and short-distance interhospital transports when being on ECMO support.

The factors that accompanied deaths were the critical condition of the patient [12, 25, 31]. The very good aforementioned results regarding transport-related mortality may result from high qualifications of team members, accurate selection of patients, type of support, means of transport, meticulous assessment, and reporting the patient to a reference center at optimal time. In our relatively small owned experience of 12 transfers, all of them were uneventful.

#### 4.4.7. Adverse Events

Unfortunately, high-risk or life-threatening situations may occur. The necessary immediate interventions must be taken within seconds and they demand highly trained personnel. In Broman and Frekman at adverse events or complications during ECMO transports, the incidence of any kind of them occurred in 31.7% cases [37]. In the recent Karolinska center paper that involved 908 transportations, the complication rate was found to be 20% [31]; Table 3 includes percentage of adverse events in single center publications [31, 37, 39].

In 6.2% critically ill subjects, more than one complication was reported [5, 37]. Most common events were related to the patient's clinical status (28.2%), of which loss of tidal volume accounted for 11.5%. Equipment-related problems can occur in 5–8%. Others were unforeseen but reported adverse events were hypothermia, intravenous lines freezing, and traffic accidents. Authors of this review in their previous paper revealed the lack of knowledge and adaptation by the ambulance service that exposed both patient and medical team to risk in 4% of the transports [6, 37]. The most experienced specialist teams dealing with critical transfers during ECMO therapy followed the procedures and protocols and created adverse events catalogs that occurred in the transport-related situations. Fletcher-Sandersjoo et al. reviewing next 586 transportation in Karolinska center have come up with the following categorization [31]:

- High risk for morbidity and mortality without response within seconds
- High risk for morbidity and mortality with no response within minutes
- Need of attention, with no risk to morbidity or mortality
- Low risk needed to be noted

Categories I and II were observed in 20% of the actions, but they had no impact on mortality (Table 3). The greatest risk (occurrence of category I and/or II adverse events) was noticed during the flights in airplane and when applying VA ECMO. Moreover, secondary transfers were associated with greater risk; therefore, special attention and a greater number of specialists should be taken. The estimated probability of adverse events was 28%. As remedial actions after the occurrence of I and II events, it was proposed to create a checklist, introduce professionals to the teams, and improve interpersonal communication.

Adverse events most often were related to such aspects as equipment failure, improper preparation (training deficiencies and insufficiencies and inadequate equipment), insufficient documentation, and suboptimal communication [31]. Despite the occurrence in 20% of transfers of situations defined as threatening within seconds or minutes (I and II), no significant statistical impact was recorded due to the observance of the rules of organization and implementation of such projects (professional, equipped team). Moreover, the review of these events resulted in a revision of the current procedure and the creation of remedial measures for the future. The issue of “tidal volume loss,” according to Broman, is debatable due to the conventional ventilation strategy. After starting ECMO, the parameters of the ventilator for lung-protection ventilation were modified, which may cause false results and may be perceived as an adverse event. The decreasing number of incidents in Karolinska team proves the increase in team skills and transport safety.

The researchers also made comparisons between groups of patients supported with ECMO and then transported vs treated with ECMO inpatient without any transfers between hospitals [28]. The reports showed no statistically significant differences in terms of survival. Salna et al. have shown that the patients cannulated by the mobile teams experienced better outcomes than those cannulated by the referring hospital medics. The fact that clinical status of individuals cannulated on-site was much worse cannot be excluded; thus, physicians must not wait for mobile team arrivals and faster actions were obligatory [16]. The type of therapy, VV or VA, also had no significant impact of eventual outcomes but subjects undergoing VA therapy can require more urgent interventions during the transfer. However, due to different indications comparisons VV versus VA ECMO applications must be done very carefully and cautiously.

### 4.5. ECMO Transportation in Poland as an Innovative Multidisciplinary Cooperation

Extracorporeal techniques in the Polish intensive care units are not routine procedures. Apart from exceptional centers in Poland where such treatment is provided due to local initiatives, there is no systemic cooperation solution in Poland that would ensure universal access to this therapy (as it requires involvement of a lot of parties, as well as unique knowledge, skills, competences, and desired experience). There are no confirmed epidemiological data regarding use (exact numbers and places) of VV-ECMO systems. More information is available on VA-ECMO therapy as they are implemented and obligatory reported to the central registry by cardiac surgical departments.

Consequently, a real need for this type transfers in Poland is difficult to estimate due to formal and organizational problems, shortages of places in higher-reference centers, and difficulties in rationally escalating the therapy, without omitting the currently strongly recommended interventions. Moreover, many individuals that would potentially benefit from ECMO support are not referred to expert centers.

Most world leading ELSO institutions have already implemented the concept of “hub and spoke,” where specialized transport of patients to the center of reference plays a fundamental role. Despite lack of dedicated ECMO centers in Poland, the Polish Guidelines issued by the National Intensive Care Consultant and VV-ECMO therapy team recommended protocols and procedures for the mobile ECMO teams' assessment, notification, and ECMO implementation before transfer that patient to the high-volume ECMO center. The “HandS” conception has been realized in 6 centers (Zabrze, Cracow, Lublin, Warsaw, Opole, and Poznan) with dedicated transportation teams. Pediatric transfer in Poland is marginal. There are individual case-study reports.

“ECMO for Greater Poland” is the nation's first regional program implementing extracorporeal support for 3.5 million inhabitants of the Greater Poland region (with Poznan the capital city) especially in patients with severe reversible respiratory failure (RRF), hypothermia, and critical states resulting in heart failure due to cardiac arrest, cardiogenic shock, or acute intoxication and promotion of donor after circulatory death (DCD) strategy in selected organ donor cases, after unsuccessful lifesaving treatment, to achieve organ recovery. It is significant that, before launching the program with dedicated transport, the ECMO support application in this region was incidental [32].

The aim of the “ECMO for Greater Poland” program is to create system-wide procedures for the identification of potential candidates for extracorporeal perfusion and their transport to specialized medical centers, in order to implement and conduct therapy at the highest possible level. It may be possible thanks to the improvement of the disposition and coordination of the Medical Rescue System, appropriate qualifications of the medical personnel of Emergency Departments and selected intensive care units, creation and specialized training of resuscitation, and perfusion and transplant teams.

**Figure 4:**
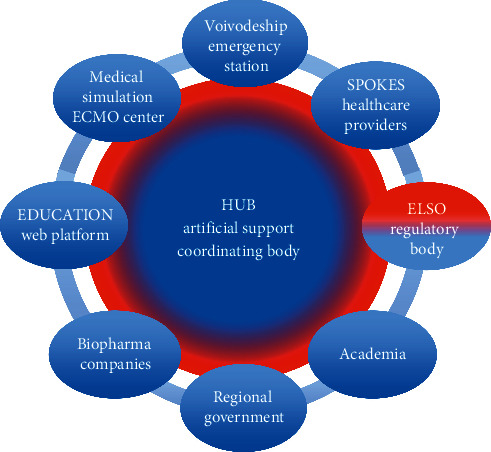
“ECMO for Greater Poland” Innovation Ecosystem.

The role of the hub is the integration of external partners, coordination of innovative cooperation, knowledge transfer and diffusion of knowledge, monitoring, management and control of the ecosystem, and continuous improvement of existing procedures, qualifications, and skills within a dedicated educational platform in order to develop mutual competencies necessary in the development of new therapies for patients [46], as shown in Figure 4. This cooperation enables a number of innovative projects and allows significant synergy effects and helps in getting easier response to changes in the environment (COVID-19 pandemic). The involvement of all partners, from both local and regional levels, from business and academia, in the cooperation, can have a positive effect on innovation cooperation performance of the whole “ECMO for Greater Poland” innovation ecosystem [47]; also, it can enable the development of new modes of cooperation [48, 49], including, for example, R&D alliances, Open Innovation alliances, cross-industry alliances, and altruistic alliances.

Since the end of the 1980s, we can observe more and more nonequity R&D alliances in the biopharmaceutical industry in the world [48], which provide greater flexibility in the selection and possible change of partners and also enable a faster change or exchange of technology than traditional equity alliances. This trend can also be seen in the region of the Central and Eastern Europe (CEE). The results of one of the first in the world qualitative and quantitative primary research focused on innovation cooperation in the biopharmaceutical industry in the CEE region, conducted within research grant entitled “Analysis of Open Innovation Alliances and Strategic Partnerships in the Biopharmaceutical Industry in Poland and CEE Countries,” showed that over 80% of companies (*n* = 107) from the biopharmaceutical industry from 18 CEE countries carried out mainly R&D nonequity alliances in the development of innovation cooperation in years 2015–2017. However, only 18% of them have implemented innovation cooperation using new modes of cooperation, Open Innovation alliances. It should be also taken into account that more than 65% of companies are open to develop cross-industry alliances and altruistic alliances (nonprofit) with companies or institutions in the biopharmaceutical industry and in other industries. With more flexible modes of cooperation, it is possible to deliver new solutions and patient treatment faster, using the innovative potential of all partners involved in the cooperation in the “ECMO for Greater Poland” program.

The ECMO mobile team was created on a voluntary basis. Thanks to the launch of the program, emergency teams in the region were equipped with 15 mechanical compression devices and one dedicated ambulance for transport of ECMO-supported patients [50]. Up to now, 12 uneventful transfers of ECMO-supported patients have been performed in the last 4 years.

It is difficult to estimate the demand for extracorporeal techniques, taking into account epidemiological needs. According to German estimates, in the corresponding population, the number of cases of severe RRF may reach 8–10 patients per million inhabitants per year. This is a significant number of patients who can be treated by applying these techniques and in the experienced centers the survival rate can be as high as 70% [51]. Of note, the most sophisticated respiratory techniques may be associated with only 10–20% survival rate [52]. VA therapy seems to be more demanding and should be concerned for use in the states of severe cardiorespiratory failure or resuscitation of various etiologies. This may also include critically ill young patients waiting for organ transplants (heart and lung). In such situations, maintenance of vital functions in the form of ECMO should be initiated in the regional hospital and then subjects should be transported to a transplant center. Extracorporeal circulatory support techniques used in the cases of end-stage heart failure are recognized therapeutic form of mechanical circulatory support and transplantation programs as “bridge to decision” or “bridge to bridge” [53].

### 4.6. ECMO Transportation in Poland in COVID-19 Pandemic

Despite the lack of dedicated ground ECMO transportation system in Poland, the COVID-19 pandemic resulted in the creation of 5 dedicated centers for VV-ECMO support in COVID patients (Lublin, Warsaw, Cracow, Gdańsk, and Wroclaw). The developing epidemic provoked the initiative of air transportation (HEMS: Helicopter Emergency Medical Service, helicopters and planes) in Poland, and for several months transport by helicopter has been available 24 hours a day in five Polish HEMS stations. During last months, few ground and air COVID patients with ECMO support were performed. In HEMS, there is one dedicated device, Cardiohelp (Getinge, Rastatt, and Germany), which has been registered under the Polish aviation regulation (without heater).

“ECMO for Greater Poland” mobile ECMO team recently performed one transfer with COVID suspected patient with ECMO-dedicated ambulance. Transfer and ECMO implantation were properly prepared with developed checklists, including team division in “cold” and “hot” zone and personal protective equipment use. For the purposes of precise separation of units and equipment, rational use of PPE, and to prevent contamination of the equipment and transport bags, an additional vehicle (for not contaminated bags and devices) with a driver was provided. The transport went without unexpected complications.

### 4.7. Role of Translational Simulation in ECMO Transportation

Translational simulation is a term describing the part of simulation activities focused on improving healthcare processes and outcomes [54]. It can be realized through diagnosing safety and performance issues as well as delivering simulation-based intervention, irrespective of the location, modality, or content of the simulation. Translational simulation can be an improving safety in ECMO transportation. We must remember that result depends on the frequency and preparation of the team and the circumstances under which the patient transfer occurs. High-fidelity medical simulations give opportunity to create and test procedures and checklists developing situational awareness and entrusting such tasks to specialized teams, prepared and trained in a given field [49].

In support of the validity of the simulation education in ECMO transfers, a review of the methods used in the world was the part of Broman et al.'s survey [39]. 14 of 15 centers performed some kind of structured training: five (33%) combine ECMO clinical and ECMO transport training (i.e., simulations and rescue training aircraft); nine centers (60%) perform regular ECMO simulations and 3 centers (20%) perform annual transport simulations. 4 others perform ECMO pump training and in-house simulations. An overview of identified simulation techniques includes the following:

- high fidelity simulations - ground and air;
- wet labs, sims;
- not formal certified workshops;
- online trainings and no sim in vehicles;
- aircraft in vehicle sim - yearly training;
- ECMO transport simulation in vehicle sim - twice in year.

The “ECMO for Greater Poland” program uses, as a superior, proprietary tool allowing the flexibility to create previously nonexistent procedures, high-fidelity simulation. It allows for high-quality personal and procedural training in an accessible and repeatable way. In the case of rare, complicated, and expensive procedures, it allows for “probing” standardized and repeated training, skills' improvement, and their verification. In addition, it allows for improving Healthcare Service, communication for best patients' outcomes [53]. The role of medical simulation in the educational process is invaluable and still underrated. The economic result of simulation training is an optimized cost of improving theoretical and practical skills.

In “ECMO for Greater Poland” program, high-fidelity simulation scenario of patient (mannequin) transfer with implanted ECMO console for mobile ECMO team was created. A 80 km transfer via road ambulance between two cities was performed. During transportation, all critical patient parameters were under meticulous control. Moreover, the efficacy of electrical sources and oxygen supply mandatory for the complex procedure was also confirmed. This probing simulation allowed us to control the deployment of qualified medical personnel and to know about necessary equipment in the ambulance. In addition, proprietary equipment lists and checklists for the individual stages of transport were created. In addition, the experience of first 5 transports in the standard ambulances allowed for designation and raised funds for the container ambulance dedicated for ECMO transport (the first ECMO ambulance in Poland).

### 4.8. Artificial Life Support with ECMO

“ECMO for Greater Poland” also has an educational intention and we have developed a course about “Artificial Life Support with ECMO” and created the “Center of Artificial Life Support and Patient Safety” within a University Medical Simulation Center. The project will be implemented in 2019–2021 at the Medical University of Karol Marcinkowski in Poznan. The project was awarded funding from a POWER competitive national grant (POWR.05.04.00-IP.05-00-006/18) by the Polish Ministry of Health for a total of 2,750,000 USD (PLN 10,974,708.60).

The program is offered to 264 physicians from Poland specializing in anaesthesia and intensive care, cardiac surgery, cardiology, thoracic surgery, vascular surgery, transplantology, and emergency medicine and other physicians in training from all over Poland. An important part of educational 3-day program is the ECMO transportation subject with lectures, transfers checklist creation, and intra- and interhospital transfers simulation scenarios. Since 2019, 154 physicians from Poland have finished the ELSO endorsed course. Moreover, 2 in 10 trained simulation scenario lists include intrahospital and interhospital ECMO patient transfer in ambulance vehicle training in Center of Medical Simulation.

ECMO team and ECMO mobile time of “ECMO for Greater Poland” program offer training twice in year interhospital ground transfer in vehicle simulator in the Center of Medical Simulation.

### 4.9. Controversies in Previous Publications and Limitation

There is a problem with accurate estimation of the ECMO transfer numbers, deaths, or undesired incidents on the basis of previously published reports because they often counted also conventional transportation. The authors reviewed the publication critically, selecting from a given center reports with most actual and containing the highest number of transports performed. In addition, the meta-analysis eliminated conventional transports from the total number.

This work does not include the transport of ECMO-supported patients with symptomatic COVID-19. All included reports had been published before the end of 2019.

We believe that this is the first analysis that captures previously repeated errors in the estimated number of transports and deaths. There should be an extended analysis of the hospital survivors of transferred patients with ECMO support following on from this.

## 5. Conclusions

The ECMO transportation of critically ill patients is a complex demanding procedure and process but it can be performed safely by well-trained and cooperating dedicated ECMO team. Key aspects of transportation include the importance of “ground time” in primary transfers, dedicated vehicle, and dedicated ECMO mobile team. Checklists and standard operating procedures are important to create safe environment for the patient and team. The valid ELSO recommendations regarding ECMO transportation are important guidelines. However, in each center it is necessary to develop individualized transfers protocols. Due to its complexity, involvement of many medical professionals, and numerous possible serious adverse events, the role of “probing” medical simulations to check on regular basis the personnel qualifications is extremely important to ensure safety of transfer of ECMO-supported individuals. We have therefore leveraged on a HUB innovation ecosystem to support the safety of the treated patients with extracorporeal support.

## Figures and Tables

**Table 1:** Summary of our experience with transfers of the ECMO-supported patients.

No.	Sex	Age (years)	ECMO type	Cannulation	Vehicle	On ECMO transportation distance (km)	Indication	Deaths or incidents
1	F	56	VV	Percutaneous	AC	120	RRF	—
2	F	19	VV	Percutaneous	AC	10	RRF	—
3	M	52	VV	Percutaneous	AC	10	RRF	—
4	M	59	VV	Percutaneous	AC	40	RRF	—
5	M	28	VV	Percutaneous	AC	50	RRF	—
6	M	2	VV	Percutaneous	AD	200	RRF	—
7	M	21	VA	Surgical	AD	80	CS	—
8	M	63	VV	Percutaneous	AD	120	RRF	—
9	F	23	VV	Percutaneous	AD	5	RRF	—
10	M	19	VV	Percutaneous	AD	10	RRF	—
11	F	38	VV	Percutaneous	AD	100	RRF	—
12	F	62	VV	Percutaneous	AD	5	RRF	—

^*∗*^Median with range (minimal/maximal values); AC: conventional ambulance; AD: ECMO-dedicated ambulance; CS: cardiogenic shock; ECMO: extracorporeal membrane oxygenation; RRF: reversible respiratory failure; VV: venovenous; VA: venoarterial.

**Table 2:** List of included studies. General information, reported transfers, vehicle type, incidents and deaths, distance, and ECMO team persons.

Author	Year of publication	Center	Reported period	No. of transfers	Transfers (month)	Transfer types		Vehicle (%**)**		Incidents (%)	Deaths	Distance (km)		Type of support	No. of ECMO team persons
						Primary	Secondary	Ground	Air			Min	Max		
Roch A. et al. [11]	2013	Marseille	48	85	1.8	85	0	100	0	0	0	xx	xx	VV	3
Bryner B. et al. [12]	2014	Michigan	240	221	0.9	221	0	69	30	13	1 (*R*) 5 (*N*)	xx	xx	VA, VV	3
Sherren P.B. et al. [13]	2015	London	12	47	3.9	47	0	98.4	1.6	2	1 (*N*)	3.7	550.3	VV	3
Vaja R. et al. [14]	2015	Leicestershire	60	102	1.7	102	0	77	22	1	0	5.8	1576.8	VA, VV	4
Raspe C. et al. [15]	2015	Halle	36	36	1.0	36	0	33	64	0	0	3.5	115.0	VV	3
Salna M. et al. [16]	2017	NY Presbyterian	105	222	2.1	175	47	98.2	1.8	0	0	3.7	11398.2	VA, VV	6
Uribarri A. [17]	2017	Salamanca	33	9	0.3	2	7	100	0	0	0	126.0	224.0	VA, VV	4
Yeo H. J. et al. [18]	2017	Yangsan	46	18	0.4	18	0	100	0	0	0	26.0	408.0	VA, VV	5
Guenther S.P.W. [19]	2017	Munich	40	40	1.0	40	0	72.5	27.5	0	0	5.6	116.4	VA	2
Cianchi G. et al. [20]	2017	Florence	87	91	1.0	91	0	100	0	0	0	5.0	407.0	VV	4
Mendes P. V. et al. [21]	2017	São Paulo	48	7	0.1	7	0	86	14	xx	xx	0.5	163	VV	4
Blecha S. et al. [22]	2018	Regensburg	20	52	2.6	52	0	67.8	32.2	11.8	0	23.0	105.0	VA, VV	3
MacDonald M.D. et al. [23]	2018	Philadelphia	36	79	2.2	75	4	39	61	1.0	1 (N)	4.8	165.7	VV	4
Heuer J.F. [24]	2018	Bochum	60	75	1.3	75	0	55	45	0	0	24.4	207.6	VV	3
Brechot N. et al. [25]	2018	Paris	48	118	2,5	118	0	100	0	7.6	1 (R)	6	25	VA, VV	2
Austin D. E. et al. [26]	2018	Sydney	108	164	1.5	130	34	58	42	7.3	0	16.0	1908.0	VA, VV	2
Dalia A.A. et al. [27]	2019	Boston	84	51	0.6	0	51	100	0	xx	xx	xx	xx	VA	xx
Ehrentraut S.F. et al. [28]	2019	Bonn	120	126	1.1	126	0	85	15	6.3	4 (N)	7.0	548.0	VA, VV	2
Bonadonna D. et al. [29]	2019	Durham	24	132	5.5	132	0	100	0	0	0	xx	xx	VA, VV	4
Wilhelm M.J. et al. [30]	2019	Zurich	88	58	0.7	49	9	40	60	0	0	3.0	225.0	VA, VV	3
Fletcher-Sandersjoo A. et al. [31]	2019	Karolinska	78	908	11.0	800	108	40.3	59.3	20.0	2 (R)	6.9	13447.0	VA, VV	2
Puslecki M. et al. [32]	2019	Poznan	36	6	0.2	6	0	100	0	xx	0	3.0	200.0	VA, VV	4

VV: venovenous; VA: venoarterial; R: related; N: not related.

**Table 3:** Sources of adverse events according to the typology of Broman et al. along with the percentage distribution after detailed analysis of 322, 514, and 908 transfers.

Sources related to	Karolinska (322 transfers) 94 incidents [37] (%)	Karolinska (514 transfers) 206 incidents [39] (%)	Karolinska (908 transfers) 252 incidents [31] (%)
Patient	70.0	65.0	62.0
Equipment	17.0	14.6	19.0
Vehicle	7.4	12.6	13.0
Environment	3.1	1.9	2.0
Personnel	2.1	5.8	5.0

## Data Availability

The data used to support the findings of this study are available from the corresponding author upon request.

## Abbreviations

ECMO: Extracorporeal membrane oxygenation
ELSO: Extracorporeal Life Support Organization
COVID-19: Coronavirus disease
RCT: Randomized control trial
VV: Venovenous
VA: Venoarterial
HandS: Hub and Spoke
RRF: Reversible respiratory failure
DCD: Donor after circulatory death
SOP: Standard operating procedure
ACT: Activated clotting time
PEEP: Positive end-expiratory pressure
ECG: Electrocardiography
SpO2: Pulse oximetry.

## Supplementary Materials

Supplementary Materials Appendix 1: the mobile ECMO team's equipment proposal for “ECMO for Greater Poland” program.
