# Supplementary material for: Patient Safety during ECMO Transportation: Single Center Experience and Literature Review
Source: Emerg Med Int. 2021 Feb 22;2021:6633208. doi: 10.1155/2021/6633208 (PMC7920709; doi:10.1155/2021/6633208)
Supplement: Supplementary Materials — Appendix 1: the mobile ECMO team's equipment proposal for “ECMO for Greater Poland” program. [file 6633208.f1.docx]

Appendix 1. The Mobile ECMO team's equipment proposal for “ECMO for Greater Poland” program.

| Support | Equipment |
| --- | --- |
| Extracorporeal support | Oxygenator, pump, set of tubes and cannulae, priming set, device for system temperature management.  Holder for a stretcher or transport system with attachment to the vehicle structure. Transport gas cylinder.  Automated mechanical chest compression device. |
| Mechanical ventilation | Transport ventilator (reinforced structure, battery powered, transport handles, safe attachment to the structure of the means of transport) - multi-stage gas mixer, PEEP, I:E, audio and visual alarm system. Transport gas cylinder for the duration of the journey.  Battery-powered transport sucker.  Self-inflating rescue bag with PEEP valve. |
| Vital functions monitoring | Transport monitor (compact, rugged design, battery powered, carrying handle) - ECG, SpO2, temperature, invasive blood pressure measurement, audio and visual alarm system. |
| Patient transportation | Transport stretcher or transport system with increased payload. |
| Examination and cannulation | Mobile ultrasound device with convex and linear probe; sterile operating field drape package, surgical instruments, portable surgical head lamp.  Diagnostic devices for acid-base balance analysis and ACT measurement. |
| Transportation vehicle | 230V power supply capable of covering the demand for all devices with a safe backup; efficient installation of medical gases, connectors compatible with the equipment, gas reserves for a double estimated transfer time. |
| Other | Necessary small equipment and medical devices (medical backpack with instruments for supplying the respiratory tract, punctures, transfusion sets, needles, syringes, medicines, etc.) |

ACT-activated clotting time; PEEP-positive end expiratory pressure; ECG-electrocardiography, SpO2-pulseoximetry
